# Children with autism spectrum disorder produce more ambiguous and less socially meaningful facial expressions: an experimental study using random forest classifiers

**DOI:** 10.1186/s13229-020-0312-2

**Published:** 2020-01-13

**Authors:** Charline Grossard, Arnaud Dapogny, David Cohen, Sacha Bernheim, Estelle Juillet, Fanny Hamel, Stéphanie Hun, Jérémy Bourgeois, Hugues Pellerin, Sylvie Serret, Kevin Bailly, Laurence Chaby

**Affiliations:** 1 Service de Psychiatrie de l’Enfant et de l’Adolescent, GH Pitié-Salpêtrière Charles Foix, APHP.6, Paris, France; 2 Institut des Systèmes Intelligents et de Robotique, Sorbonne Université, ISIR CNRS UMR 7222, Paris, France; 3 Université de Nice Sophia Antipolis, Nice, France; 4 Institut de Psychologie, Université de Paris, 92100 Boulogne-Billancourt, France

**Keywords:** Autism spectrum disorder, Facial expressions, Emotion, Algorithm

## Abstract

**Background:**

Computer vision combined with human annotation could offer a novel method for exploring facial expression (FE) dynamics in children with autism spectrum disorder (ASD).

**Methods:**

We recruited 157 children with typical development (TD) and 36 children with ASD in Paris and Nice to perform two experimental tasks to produce FEs with emotional valence. FEs were explored by judging ratings and by random forest (RF) classifiers. To do so, we located a set of 49 facial landmarks in the task videos, we generated a set of geometric and appearance features and we used RF classifiers to explore how children with ASD differed from TD children when producing FEs.

**Results:**

Using multivariate models including other factors known to predict FEs (age, gender, intellectual quotient, emotion subtype, cultural background), ratings from expert raters showed that children with ASD had more difficulty producing FEs than TD children. In addition, when we explored how RF classifiers performed, we found that classification tasks, except for those for sadness, were highly accurate and that RF classifiers needed more facial landmarks to achieve the best classification for children with ASD. Confusion matrices showed that when RF classifiers were tested in children with ASD, anger was often confounded with happiness.

**Limitations:**

The sample size of the group of children with ASD was lower than that of the group of TD children. By using several control calculations, we tried to compensate for this limitation.

**Conclusion:**

Children with ASD have more difficulty producing socially meaningful FEs. The computer vision methods we used to explore FE dynamics also highlight that the production of FEs in children with ASD carries more ambiguity.

## Introduction

The understanding of our facial expressions (FEs) by others is crucial for social interaction. During typical development (TD), most facial components of the human expression repertoire can be observed shortly after birth (e.g. smile), even if FEs in infancy are not the same as their adult counterparts [1, 2]. First, emotion in infancy cannot be compared to emotion in adulthood because emotions at these stages do not involve the same degree of cognitive/emotional complexity [3]. Second, facial motricity is not similar between infants and adults [4]. Fully adult-like FE physiognomies appear progressively, and the learning of FEs increases even in late childhood: the ability to produce FE continuously improves between 5 and 13 years of age and 13-year-old adolescents do not yet produce all FEs perfectly [5, 6]. Their production is influenced by several factors: (1) emotional valence (e.g. positive emotions are easier to produce than negative emotions) [6–8]; (2) gender, as girls tend to produce positive emotions more easily than boys, and boys tend to produce anger more easily than girls [9]; (3) the type of task, as this factor modulates the quality of FEs in children (e.g. children are better with request tasks than with imitation tasks) [6]; and (4) ethnic and cultural factors (e.g. cross-cultural studies indicate that the intensity of spontaneous expression is not universal and may vary across cultures) [10, 11]. Similarly, in a previous study [6], we found that children from the south of France (Nice) were more expressive than children from the north (Paris).

Autism spectrum disorder (ASD) is a neurodevelopmental disorder characterized by repetitive behaviours and impairments in social interaction, including deficits in social expression that is crucial for communicating one’s internal state to others and that can impact social integration [12, 13]. A recent meta-analysis found that participants with ASD were less expressive and that their FEs were incongruous with the social context [14]. The inadequacies observed in various studies seem to be related to the way FEs are conceptualized (e.g. visual appearance, intensity or reciprocity in social contexts); operationalized, in terms of the tasks best able to elicit them (e.g. spontaneous vs. posed facial expression); and measured (rating by observers, automatic analysis using electromyographic recording or FE analysis software based on the Facial Action Coding System).

When the deficits are explored in terms of the number of spontaneous FEs produced during videotaped child-experimenter interactions, children with ASD, children with intellectual disability and matched TD children do not differ in the quantity of positive FEs produced. However, children with ASD produce more negative FEs than the two other groups [15]. When FEs are rated by neurotypical observers with a dimensional approach using Likert scales, no difference is found in FE intensity between children with ASD and TD children [16]. However, the results differ when other types of tasks are performed, such as more naturalistic tasks (i.e. spontaneous expressions evoked in social contexts) during which children with ASD may be judged to be more expressive [17, 18], even if these results are not consistent [19]. However, in most studies, it appears that children with ASD produce more ‘bizarre’ or ‘mechanical’ posed expressions [20], ambiguous FEs in child-experimenter settings [15] and ‘awkward’ FEs during emotional storytelling [16]. Atypical FEs in autism do not arise from a specific way of producing FEs but appear to be idiosyncratic: individuals with ASD and TD individuals have the same difficulty in recognizing FEs produced by individuals with ASD and are better able to recognize FEs produced by TD individuals [21]. Moreover, individuals with ASD are less able to recognize anger produced by individuals with ASD than TD individuals [21]. Additionally, individuals with ASD do not share common representations for FEs [21].

Among the factors that could influence FE production in individuals with ASD, emotional valence is key. Positive FEs are easier to produce than negative FEs [22, 23]. Sadness is the most difficult to produce in children with ASD [19], but not in adults with ASD [21, 23]. This difference between studies could be explained by differences in the methodology used, as the type of task also influences FE production in ASD [20, 21, 23]. The effect of gender on FEs in ASD has only been investigated in adulthood. It seems that males with ASD more accurately produce FEs than females, although FEs of females were rated as being more natural than those of males [23]. The social context may also modulate FEs, although the results are not consistent (see [24] for a review). In the Trevisan meta-analysis [14], spontaneous and posed FEs were both produced better by typical participants. Additionally, the accuracy of FE production seems to increase with age and intellectual quotient (IQ) in individuals with ASD [14]. We found no studies about the potential effect of ethnicity or cultural environment on FEs in individuals with ASD.

The development of affective computing and, more specifically, FE analysis from videos allows the automation of FE recognition in neurotypical adults, even with variability in lighting conditions or subject morphology, as well as the understanding of FE dynamics (e.g. [25]). In children with ASD, the first attempts to use computer vision tried to model specific characteristics of FE dynamics. Samad [26] found a significant asymmetry in the activation of specific pairs of facial muscles in adults with ASD compared to those of TD controls. This asymmetry is presented as a potential explanation for the impression of oddity in FEs produced by adults with ASD. The groups of Grossman and Narayanan showed that children with ASD had less synchrony of motion between facial regions and a higher level of variability [27] and that they displayed less complex facial dynamics (assessed by multiple scale entropy), specifically in the eye region [28]. In their last study, they also showed that distance features were less predictive of the emotional valence for FEs in ASD than in typical participants [29]. Combining recorded videos of the Autism Diagnosis Observation Schedule (ADOS) and commercially available software, Owada et al. [30] found that individuals with ASD produce significantly fewer variations of neutral and joyous FEs than TD individuals. However, these promising preliminary studies had several limitations: limited sample size, only high-functioning adult males with ASD, no assessment of possible factors that could influence children’s productions (e.g. age, type of task, social context) and no use of machine learning algorithms due to the lack of access to a large database of children’s FEs.

There are multiple rationales for using machine learning algorithms for analysing facial expressions: (1) to obtain deep insights into what kind of features are relevant when children are producing a given facial emotion due to the investigation of the most relevant features that have been selected by the algorithm, (2) to explore large video datasets of children and (3) to highlight objective differences between ASD and TD children productions based on the capture of relevant features (e.g. distance between facial landmarks [29]). In comparison, motion capture allows a higher temporal resolution and can capture micro-expressions but brings only geometric information. In addition, motion capture is more invasive as it requires the use of specific sensors on the child’s face.

We only found one study showing the potential value of an FE recognition pipeline [31]. This study proposed a computational approach combining the exploration of FEs in unconstrained conditions, the estimation of action unit intensities by analysing local appearance and the use of machine learning classifiers. The outputs were compared with evaluations performed by expert raters on a group of 17 children with ASD. The results showed how this computational assessment of FE dynamics helped go beyond the traditional qualitative rating, which may be affected by human limitations, for observing subtle multi-cue behaviours [31]. However, this study also had limitations: small sample size of children with ASD and the use of an adult database to train the classifiers despite the well-known differences between adults’ and children’s FEs [3].

The use of automatic FE analysis to characterize FE production in ASD appeared promising. However, many questions remain regarding FE production in children with ASD, as most studies have been conducted in adult/mixed populations. Additionally, the development of FE production in children with ASD is not well understood, nor are the factors that could influence this process [14]. Our work pursues two aims:

(1) Investigating FE production in children with ASD who are 6 to 12 years old, comparing their production to that of TD children, taking into account different factors that could influence FEs [6]. Based on previous research, we hypothesized that FE quality would increase with age and with IQ, that positive emotions would be easier to produce than negative emotions, that the type of task would influence the children’s production, that the FEs of children from Nice would be rated with more accuracy than the FEs of children from Paris, that gender would influence the children’s productions, that TD children would produce more accurate FEs than children with ASD and that children with ASD would be helped by the presence of a model.

(2) Exploring, with random forest (RF) classifiers, how children with ASD differ from TD children during FE production. In other words, the RF classifiers behave as automated expert raters, offering the opportunity to explore how they reach their classification. We extract geometric and appearance features because they are complementarity and are often used in the automatic facial expression recognition domain [32]. Geometric features characterize facial landmark displacement (e.g. a smile), while appearance features can encode information such as the occurrence of wrinkles. We hypothesized that (1) the RF classifiers would reach a better accuracy with typical children than with ASD children and that (2) children with ASD would specifically use their mouths to produce FEs as a difference between the interest in the mouth and the eyes during emotional recognition has previously been shown [33].

## Methods

### Participants

We enrolled 157 TD children between the ages of 6 and 11 years old (*N*_boys_ = 52%). All TD children were recruited in two schools, one in Paris and one in Nice. All participants were native French children, and the same numbers of children were recruited by grade. We also recruited 36 children between the ages of 6 and 12 years old (*N*_boys_ = 75%) with a diagnosis of ASD as confirmed by at least one validated method (ADOS and/or Autism Diagnosis Interview-Revised, ADI-R). The children were recruited from two hospitals in France, one in Nice (*N* = 20) and one in Paris (*N* = 16). The participants’ clinical characteristics are summarized in Table 1. Before inclusion, written consent was obtained from parents and children after they were given proper information. The researchers met with each child alone for approximately 40 min to complete the protocol. The study was approved by the ethical committee of Nice University (*Comité de Protection des Personnes Sud Méditerranée*) under the number 15-HPNCL-02.

**Table 1:** Main characteristics of the participants

	ASD (*N* = 36)	TD (*N* = 157)
Chronological age, mean (±SD)	8.8 (1.8)	8.4 (1.4)
Male/female (% of males)	27/9 (75%)	82/75 (52%)
Nice/Paris (% from Nice)	20/16 (55.6%)	94/63 (60%)
WISC-4, mean (±SD)	92.5 (17.5)	Not performed
Developmental age (IQ × age/100)	8.2 (2.1)	NA
ADI-R scores, mean (±SD)		
Social impairment	14.9 (5.1)	NA
Verbal communication	11.9 (6)	NA
Restricted, repetitive behaviours	4.7 (3)	NA

### Tasks

The protocol is available in detail in Grossard et al. [6]. It consists of two tasks of FE production, one entailing a verbal request without a model (the on request task) and one entailing the imitation of an avatar (the imitation task). In each task, the child must produce FEs only (visual modality) or both facial and vocal expressions (audio-visual modality). The order of the two tasks was counterbalanced across each modality, resulting in four orders of presentation for the tasks. Children had to produce four FEs: joy, anger, sadness and neutral expressions.

**Fig. 1:**
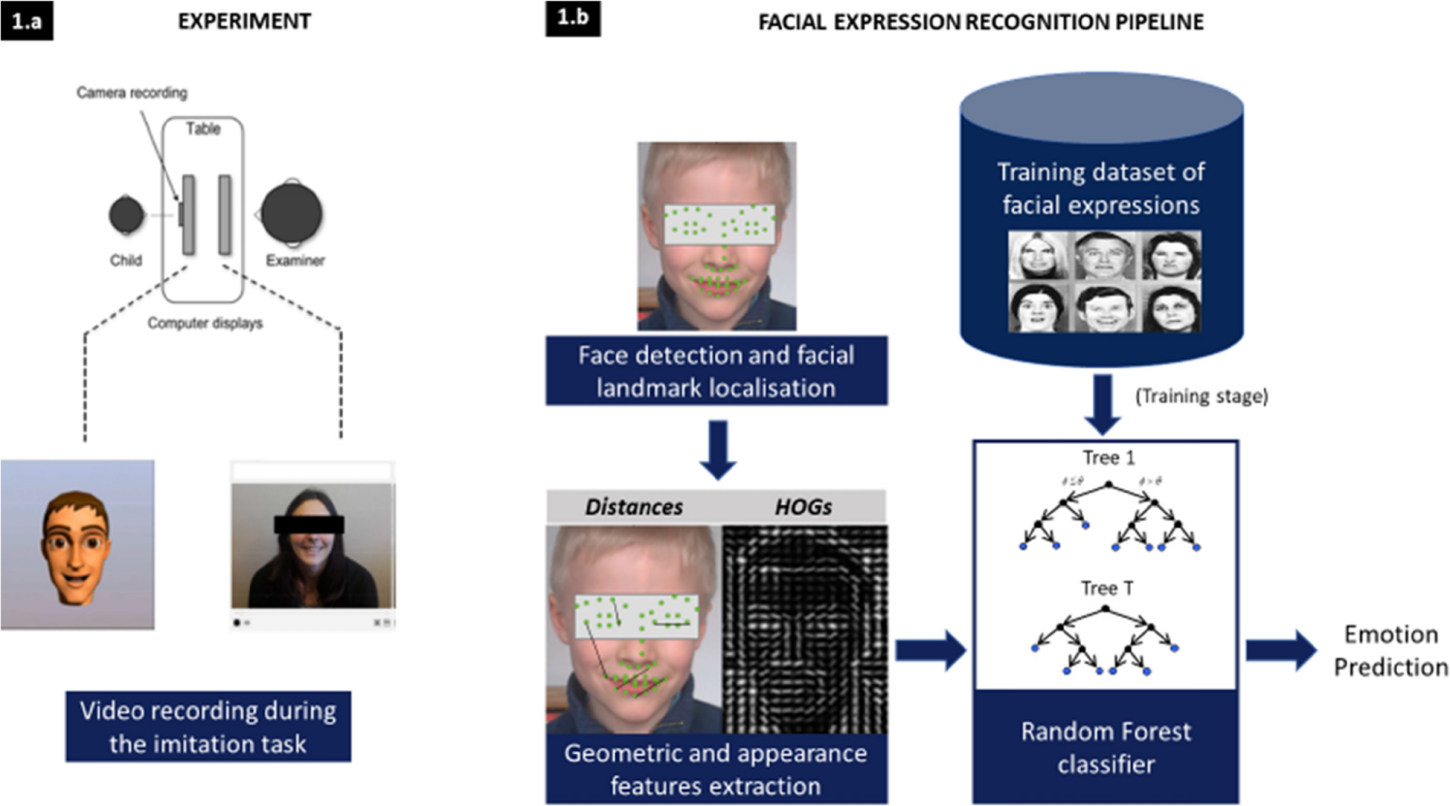
Framework of the computer vision method to explore FEs in TD children and children with ASD: experiment to induce FEs (**a**) and FE recognition pipeline (**b**)

In the imitation task, the child must imitate the facial productions (visual modality) and the facial and vocal productions (audio-visual modality) of an avatar presented on the screen in short 3–4-s videos (Fig. 1a). The audio-visual condition combines FEs with emotional noises (such as crying for sadness, rage for anger or pleasure for joy and a sound held for neutral emotion). These sounds were extracted from an audio dataset validated in adults [34]. The following instructions were given:

- [Visual modality]: ‘You will see an animated face on the screen. It will produce an emotion with its face, such as joy, for example. You will have to do the same thing with your face.’
- [Audio-visual modality]: ‘You will see an animated face on the screen. It will produce an emotion with its face and his voice, such as joy, for example. You will have to do the same thing with your face and your voice.’

We used one male and one female avatar for this task. The avatars and the FEs were presented in a random order. Each avatar produced one of the four FEs in both modalities (visual and audio-visual), allowing us to collect 16 videos per child.

In the ‘on request’ task, the child had to produce a facial expression (visual modality) or a facial and vocal expression (audio-visual modality) upon request. The name of the emotion was displayed on the computer screen and was read by the clinician. The following instructions were given: ‘I will tell you a word that expresses an emotion when we feel something: - [visual modality]: Could you show with your face what you do when you feel sadness / joy / anger / nothing?

- [audio-visual modality]: Could you show with your face and your voice what you do when you feel sadness / joy / anger / nothing?’

The order of the presentation of emotions within this task was also random. We presented the four FEs in both modalities (visual and audio-visual). In total, 24 (3 × 8) videos per child were collected during the entire protocol.

We first met all TD children in their school and studied the factors that influence their productions [6]. The following year, after developing the FE recognition algorithm (see below), we then met all the ASD children at hospitals.

### Judge ratings

To analyse the FEs produced by the children, all videos recorded needed to be annotated. Few studies attempt to rate the quality of emotions, and the optimal methods have not been agreed upon. In children’s studies, Egger et al. [35] asked judges how well the emotion was portrayed. Mazurski and Bond [36] looked at the certainty of the judge that the emotion he recognized was the good one. In adult studies, such as the GEMEP (Geneva Multimodal Emotion Portrayal) [37], judges had to rate the authenticity and the plausibility of each FE. For our purpose, we used a Likert scale combining two dimensions: recognisability and credibility, where we assumed that (1) an emotion can be recognized but not credible, but that (2) the opposite (credibility without recognizing the emotion) is not possible [6]. The credibility can be defined as ‘how much do you think that the children effectively feel this emotion.’ The scale allowed us to judge the presence of a given emotion on a 10-point gradient (0 = no recognition, 5 = recognition is maximal without credibility and 10 = both recognition and credibility are maximal). The score represents the quality of the FE. For each video, the judges had to complete four scales (one for each emotion: happiness, sadness, anger and neutral). This method allowed the judge to annotate one to four emotions for a given FE watched on a video. Indeed, a perfect production of happiness would be rated 10 on the scale for happiness and 0 on the three other scales. However, for a less-specific expression (such as when children laugh when trying to produce anger), the judges would annotate multiple emotions for a unique expression (such as anger 5 and joy 5). This method allowed us to annotate ambiguous FEs. We asked three judges to annotate each video of each child with ASD. They could look at the video as many times as they wanted and were also asked to indicate the beginning and the end of the FE. For videos recorded in the audio-visual modality, judges rated videos with both images and audio. The judges were French Caucasian adults (5 women) aged 22, 23, 25, 25 and 34 years. Two were students in speech therapy, two were speech therapists and one was a developmental psychologist. They were all part of the team and knew about the purpose of this research and the diagnosis. The videos were rated on the same special tool created for our preceding study on typical children FEs. Inter-rater agreement on 240 videos from 10 TD children (one girl and one boy for each age) was assessed using intraclass correlation coefficients. We found excellent agreement between the judges (ICC_happiness_ = 0.93, ICC_anger_ = 0.92, ICC_sadness_ = 0.93, ICC_neutral_ = 0.93) [6]. We also calculated the ICCs on 329 videos of children with ASD (corresponding to 20 children) and found very good agreement between the judges (but less than for the TD children) (ICC_happiness_ = 0.82, ICC_anger_ = 0.82, ICC_sadness_ = 0.79, ICC_neutral_ = 0.79). These differences support the idea that FEs produced by children with ASD are harder to classify. To enhance automatic learning, we kept only videos rated above 7 for the targeted emotion (see below).

### Statistical analysis of judge ratings

All statistics were performed using R. To analyse judge ratings for FEs, we performed generalized linear mixed models (GLMM) without interaction to assess the effect of different variables on the score obtained on our FE quality scale. The independent variables were age, gender, IQ, order of presentation of the tasks (four different orders), modality (visual vs. audio-visual), emotion subtype (anger, joy, sadness or neutral expression), centre (Nice vs. Paris), and group (ASD vs. TD). We used a second model to explore the interaction between group and emotion subtypes because the results are contradictory in the literature (see the ‘Introduction’ section).

### Pre-processing for the extraction of FE features

For each video, we applied an OpenCV Viola & Jones face detector [38] on the first frame, which had been converted to greyscale levels beforehand. Then, we applied the interface facial landmark tracker to locate a set of 49 landmarks on the face [39]. Facial landmarks correspond to semantic points localized on the face such as the mouth and eye corners and nose tip. These landmarks encode geometric deformations of the face. Next, we tracked those landmarks in the remaining frames of the video. Because the end of each video usually depicted the apex of the emotion, we selected the last frame of each video to train and test the facial expression recognition models. We rejected some videos for which the feature point tracker could not follow the head motion. We extracted a total of 3781 images for the TD children and 814 for children with ASD. For FE classification, we only used the examples whose quality was rated higher than 7 out of 10, resulting in a total of 1845 images for the TD group (corresponding to 48.8% of the videos recorded) and 332 for the group of children with ASD (corresponding to 40.8% of the videos recorded). No child was excluded during this step with 2 videos minimum to 23 videos maximum per child. This variation between children was the same in children with ASD as TD children. Finally, for better computation, all images were rescaled to a constant size of 256 × 256 pixels.

### Automatic annotation of FEs using computer vision machine learning

A traditional FE recognition pipeline consists of two different parts: feature extraction and classification (Fig. 1b). During the feature extraction step, we used the extracted facial landmarks to generate a set of features that could usually be distinguished as one of two types: geometric ones and appearance ones. We extracted 1617 features, including 1176 geometric features and 441 appearance features. For the geometric features, we decided to compute the distances between each landmark position and normalize those distances by the inter-ocular distance (IOD) for in-plane rotation and scale invariance. For appearance features, we used a histogram of oriented gradients (HOG), a feature descriptor that is known for its descriptive power and robustness towards illumination changes [31]. We computed horizontal and vertical gradients for a window of 20% of the IOD around each facial landmark. Then, those gradients were used to generate 9 feature maps per feature point, the first containing the gradient magnitude, and the remaining 8 corresponding to an 8-bin quantization of the gradient orientation [40].

Next, the classification task was performed. Random forest (RF) classifiers are a popular learning framework introduced by Breiman [41]. This framework has been used often in computer vision because it handles very high-dimensional data (such as images) and can be easily parallelized for fast training and evaluation. We used the scikit-learn implementation of the RF classifier constructed with 500 trees, a maximum depth of 16, Gini entropy for the impurity criterion, a maximum of 220 features for each split node and a minimum of 40 examples to split a node. We also used class weight balancing because of the highly skewed label distribution, as shown in Table 2.

**Table 2:** Distribution of emotion subtypes according to groups (TD vs. ASD)

Expression	TD (%)	ASD (%)
Neutral	*N* = 677 (36.5%)	*N* = 125 (37.7%)
Happiness	*N* = 525 (28.5%)	*N* = 101 (30.4%)
Anger	*N* = 397 (21.5%)	*N* = 58 (17.5%)
Sadness	*N* = 246 (13.5%)	*N* = 45 (14.4%)

We used a 10-fold subject independent cross-validation to assess the performance of our RF classifiers in the classification task in three different cases: (a) learning from TD children and testing on TD children, (b) learning from children with ASD and testing on children with ASD, and (c) learning from TD children and testing on children with ASD. Because we wanted to show that the differences in performance between (a) and (c) do not depend on the statistical repartition (number, age, city, gender) of the children, we also managed to find two subgroups of TD children, namely, TD group 1 and TD group 2, whose statistical repartition was almost identical to that of the ASD group in terms of age, gender, city of recruitment and number of videos for each targeted emotion (see Additional file 1). Thus, we were able to train two more RF classifiers for which the training was performed with the complementary TD group of TD group 1 and was tested on TD group 1 and the ASD group; the same thing was done for TD group 2.

Finally, although the automatic learning was based on vision computing, we also investigated whether the duration of the FE could differ from one emotion to another and between TD children and children with ASD (see Additional file 1).

## Results

### Rating of FEs by human judges

**Fig. 2:**
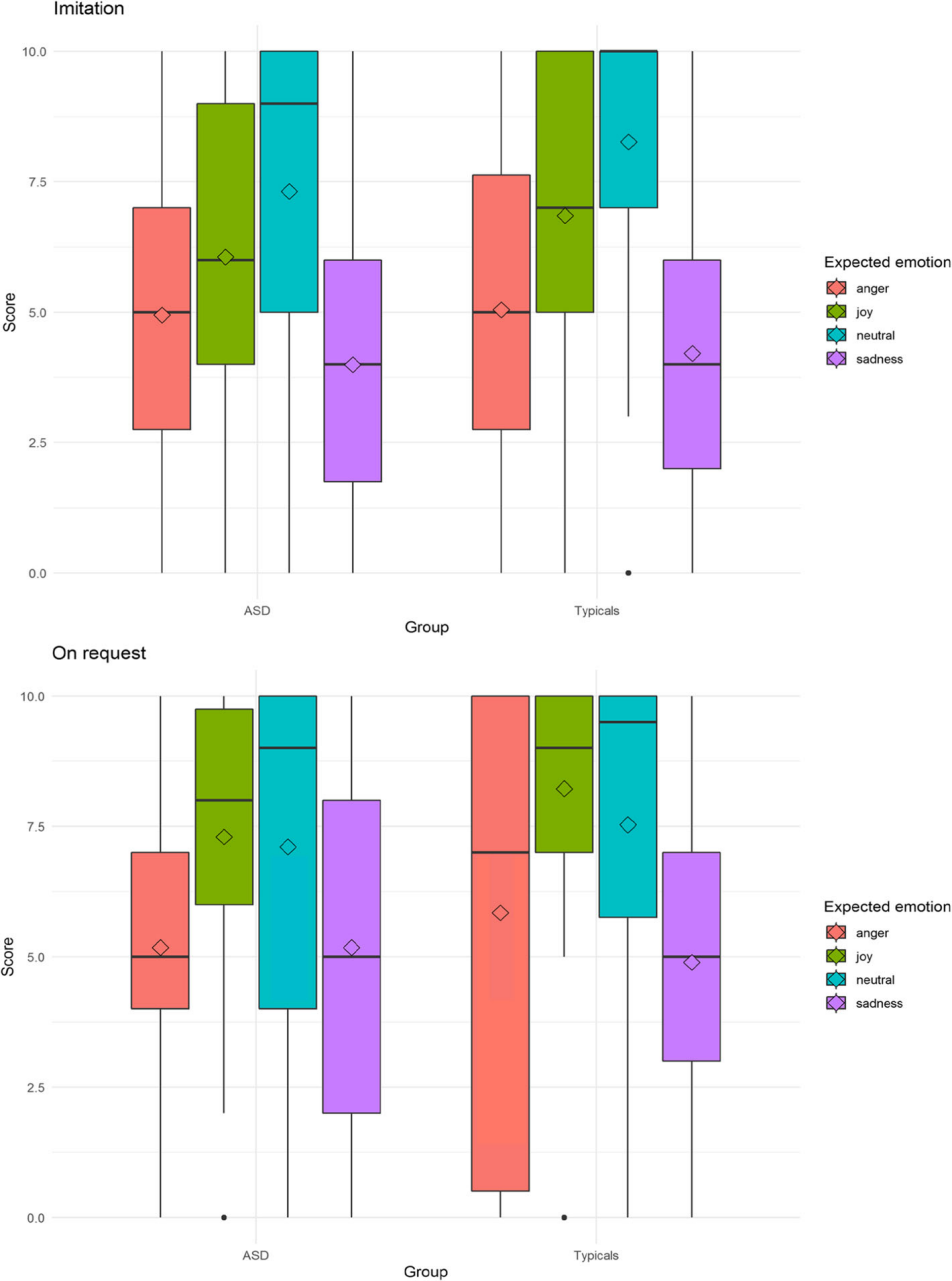
Boxplots: the solid line represents the median of the distribution; the outlines of the box represent the interquartile range, or 25th–75th percentiles; the whiskers represent the upper and lower quartiles, excluding outliers; and the diamonds represent the mean

Overall, children with ASD were able to perform the tasks with some success. Figure 2a and b show how children with ASD and TD children performed the tasks (a for imitation; b for on request) according to age and emotion subtype.

As explained in the statistics section, we performed a multivariate analysis to assess possible group differences in the scores obtained on our FE quality rating scale, taking into account other explicative variables such as age, gender, order of task presentation, modality, emotion subtype and centre location (Nice vs. Paris). The GLMM formula was the following: score ~ age + gender + order + task + modality + emotion subtype + centre location + group + (1|child number). Table 3 summarizes the GLMM statistics. Children with ASD had more difficulty producing FEs with an emotional valence than TD children. Better emotion production scores were obtained during the ‘on request task’ than during the ‘imitation task’. Emotion production scores were higher in the children recruited in Nice, and positive expressions were easier to produce than for negative expressions. Within negative emotions, anger was easier to produce than sadness. The neutral expression was the easiest FE to produce. Finally, older children tended to be better at producing FEs (*p* = 0.084). To explore why age did not reach statistical significance, we not only explored the same model for ASD children but also included the IQ variable. The model was grossly the same, but IQ was significant (see Additional file 1: Table S2).

**Table 3:** Emotion production in TD children and children with ASD as a function of age, gender, group, order, modality, elicitation task, emotion and test site: results from the GLMM model

Variable	ß estimate	Standard error	*p*
Age	0.06	0.037	0.084
Gender (boys vs. girls)	− 0.004	0.11	0.97
Order 2 vs. order 1	− 0.07	0.15	0.64
Order 3 vs. order 1	− 0.09	0.15	0.56
Order 4 vs. order 1	− 0.05	0.15	0.76
Modality (visual vs. audio-visual)	0.11	0.07	0.12
Elicitation task (on request vs. imitation)	0.54	0.07	< 0.001
Emotion (happiness vs. sadness)	1.45	0.1	< 0.001
Emotion (neutral vs. sadness)	1.63	0.1	< 0.001
Emotion (anger vs. sadness)	0.86	0.09	< 0.001
Site (Paris vs. Nice)	− 0.32	0.12	0.009
Group (typical children vs. ASD)	0.363	0.124	0.004

Since the most difficult emotion to produce appeared to be sadness, we calculated the model adjusted odd ratios with sadness as the referential emotion. The FE rating significantly increased by a factor of 2.36 for anger, 4.27 for happiness and 5.11 for neutral compared to that for sadness. The FE score significantly increased by a factor of 1.71 for the on request task compared to that of the imitation task. The FE score significantly decreased by a factor of 0.73 for children from Paris compared to that for children from Nice. Finally, the FE rating score for TD children increased by a factor of 1.44 compared to that for children with ASD.

To assess whether the duration of videos could be a confounder, we detail the duration data of the recorded videos in Additional file 1: Table S3. As shown, durations were significantly different from one emotion to another (anger≈joy < neutral≈sadness) and between tasks (avatar imitation < on request), groups (ASD < TD) and modalities (multimodal < unimodal). No single pattern emerged: the best-rated emotion (joy) was the second shortest, whereas videos of TD children (who were better rated) lasted longer than videos of children with ASD. In addition, the relevance of video duration (mean = 92.03 ± 37.38 ms) may be questionable, as shown in the distribution. None of the significant differences in video durations were above 1 SD (Additional file 1: Figure S1). Finally, when we added video duration into the GLMM, we found that shorter videos tended to be better rated than longer videos, but this effect was not significant (*p* = 0.082). The odds ratio corresponding to an increase in the duration of a video by 37 ms (corresponding to one standard deviation) was equal to 0.88. This effect was adjusted for the emotion type.

Finally, we tested the interaction between the variables – group and emotion subtypes. The GLMM formula was the following: score ~ age + gender + order + task + modality + emotion subtype + centre location + group + group × emotion subtype + (1|child number). However, we found no significant interaction between these two variables (Table 4).

**Table 4:** Interaction model between group and emotion with sadness as the referential emotion modality

Variable	ß estimate	Standard error	*p*
Emotion (happiness) × group (ASD vs. typical children)	− 0.062	0.249	0.803
Emotion (neutral) × group (ASD vs. typical children)	0.309	0.248	0.212
Emotion (anger) × group (ASD vs typical children)	0.284	0.300	0.216

### Rating FEs with a computer vision algorithm

Table 5 summarizes the recognition accuracies of our RF classifiers according to groups and emotion subtypes. For the three presented combinations of training and testing, the trained models output better accuracies when training and testing were performed on TD children. Additionally, the trained models output better accuracies for neutral and happiness classes and worse accuracy for sadness. Indeed, sadness is the more subtle FE, and the behavioural results yielded the same results (see Table 3). However, the low number of examples could explain why the RF classifiers did not efficiently capture the variability in describing sadness FEs. When training and testing were performed on ASD children, the model had poorer performance. If the algorithm did not perform similarly for TD and ASD individuals, it is because FEs produced by children with ASD are more ambiguous than those of TD children. It is important to note that the TD database outsizes the sample of ASD individuals. Therefore, the algorithm trained on ASD faces has fewer examples to train with, which could explain why generalization is more difficult. However, when training was performed on TD children and testing was performed on children with ASD, the model did not generalize well and had similar results to the model that trained and tested on children with ASD. A notable exception is happiness (grey cells in Table 5). Happiness was recognized better in children with ASD when the model was trained on TD children than on children with ASD, thus suggesting that happiness is produced by TD children and by children with ASD in a similar way.

**Table 5:** RF classifier accuracy recognition of FEs by cross-validation

Learning on	TD all (*N* = 157)	ASD (*N* = 34)	TD all (*N* = 157)
Testing	TD all (*N* = 157)	ASD (*N* = 34)	ASD (*N* = 34)
Neutral	86.64	72.55	68.08
Happiness	90.47	70.38	85.05
Anger	79.76	58.17	58.62
Sadness	56.15	41.79	44.44
Global accuracy (SD)	82.05 (0.08)	66.43 (1.57)	69.3 (4.62)

To control for the possible contribution of gender, age and city origin in the group differences, we selected two subgroups of TD children (TD group 1 and TD group 2) with a similar distribution of gender, age, city and proportion of available videos as children with ASD for each emotion (see Additional file 1: Table S1). In the next analysis, the RF was trained on the TD children who were not in TD group 1 or TD group 2 and was tested on the ASD group, TD group 1 or TD group 2. Additional file 1: Table S4 shows that the results of training on TD children and testing on TD group 1 or TD group 2, as well as training on TD children and testing on ASD children, are similar to the one presented previously. Thus, the statistical repartition does not seem to affect the results of the RF classification.

To ensure that those differences were not caused by the differences in the sample sizes, we trained two random forests with a 10-fold cross-validation on only TD group 1 and TD group 2. The results are shown in Additional file 1: Table S5. The changes in the sub-dataset sizes only affected the performance of sadness accuracy. This emphasizes the fact that poorer learning and testing performance on ASD is not caused by a lack of data and that the comparison between the importance of each landmark is relevant.

### Exploring FE dynamics with a computer vision algorithm

**Fig. 3:**
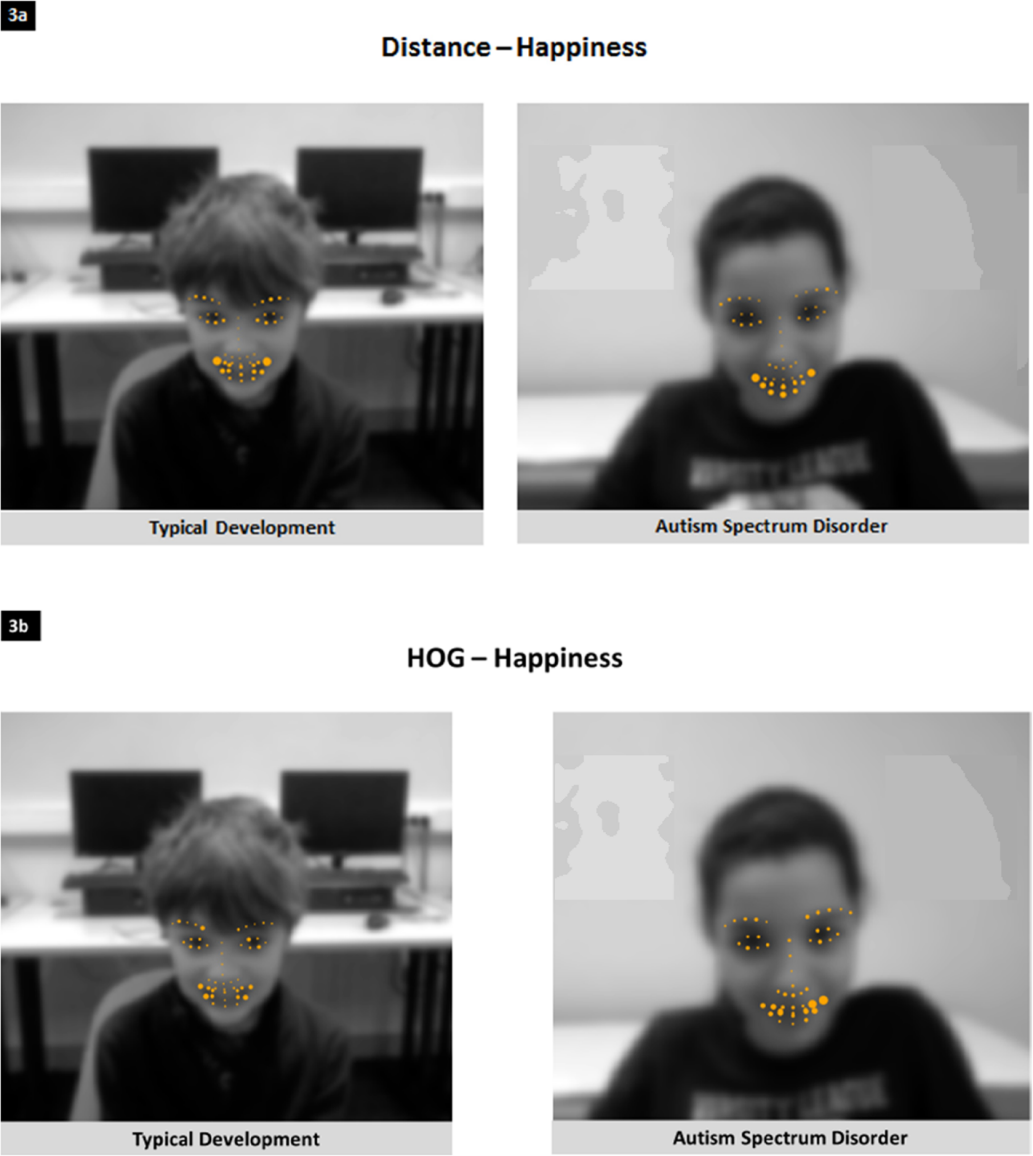
Facial landmarks contributing to the classification of happiness using RF classifiers (training and testing) in TD children (left) and children with ASD (**a** distance; **b** HOG)

To explore how ASD participants produced FEs compared to TD participants, we provide some insights on what the models learned and which facial landmarks were the most useful for discriminating between the various FE classes and qualities. These feature maps are proposed for each type of feature (geometric and appearance). Thus, the importance of a facial landmark is related to the type of feature. First, 86.7% of the classification task was based on distances (geometric features), whereas 13.3% of the classification was based on HOG (appearance features) when the RF classifier was trained on TD children. For RF classifiers trained on ASD children, the relative contributions were 83.3% and 16.7% for distance and HOG features, respectively. Examples are given in the supplemental material for anger, neutral and sadness FE (Additional file 1: Figures S2–S7). In Fig. 2, we chose to discuss happiness because this emotion was almost equally produced by children with ASD and TD children, meaning that performance could not explain why the RF classifier learned in a different manner. As shown in Fig. 3 a (distances) and b (HOG), the RF classifier needed more facial landmarks (the more a feature is needed at a specific facial landmark, the larger the point is) to achieve the best classification in children with ASD compared to TD children, in particular for facial landmarks around the mouth. Additionally, in the TD child, the mouth is symmetrical, with large points at the extremities and small points in the middle. In contrast, in the child with ASD, the symmetry is not perfect, and large points are distributed on the entire area of the lips and mouth. Regarding the upper part of the face, the RF classifier found more information (larger points) in the eyebrows and the eyes of the TD children. This was less the case for the children with ASD. It is likely that the eye regions contribute more to the recognition of happiness by the RF classifier in TD children. One possible hypothesis to explain the differences in the FE dynamics detected by the RF classifier for happiness is that the variance in the production of children with ASD is too high in the eyes region. As a consequence, the algorithm essentially used the mouth to identify happiness in the children with ASD. This difference highlights that the production of FEs in children with ASD carries more ambiguity.

Finally, ambiguity can also be captured in the confusion matrices that are shown in the supplement materials (Additional file 1: Tables S6–S10). Overall, anger expressions are often misclassified as neutral or sad expressions. Sadness expressions are often recognized as either anger or neutral expressions. This discrepancy is due to the higher level of subtlety and variability in those two FEs (i.e. anger and sadness). Moreover, when RF classifiers were tested on children with ASD, anger was also confounded with happiness. This underlines a major difference between TD children and children with ASD: in the latter, RF classifiers have more difficulty distinguishing between positive and negative expressions, meaning that children with ASD have difficulty producing FEs with clear emotional cues.

## Discussion

The two aims of our study were (1) to assess the impact of different factors (e.g. age, emotional subtype) on the production of FEs in children with ASD and to compare the production of these FEs to those of TD children and (2) to use a machine learning approach to understand which facial landmarks and features are relevant for predicting FEs and to characterize the differences in FE dynamics between TD children and children with ASD.

### Factors influencing FE production in children with ASD

The multivariate model based on children with ASD and TD children did find a series of variables we expected to influence FE [6, 14]. As expected, we found a significant group effect. The FEs of children with ASD were given lower scores than the FEs of TD children. These results are similar to those of earlier studies that found that the FEs of children with ASD were less clear and more awkward than the FEs of TD children [15, 16]. Additionally, FE emotional valence that has been shown to be an important variable in TD children [6] was also an important variable in children with ASD as the model remained significant when we added children with ASD to the model. Neutral expressions are the easiest to produce. Positive expressions (joy) are more easily produced than negative expressions (anger and sadness). Sadness is the FE that was rated with the lowest quality. The study of potential statistical interactions between groups and emotional valence (Table 4) shows that the deficit in FEs found in ASD children appears to be independent of the emotion subtype. These observations join the conclusions of the study of Brewer et al. [21] on adults and part of the conclusion of Volker et al. [22], who found that positive emotions were easier to produce than negative emotions in children with ASD. Regarding sadness, we did not find a specific deficit, in contrast to Volker et al. [22], but it should be noted that sadness was the most difficult FE to produce in both TD children and children with ASD. In addition, for all FEs, the gap between the scores of TD children and those of children with ASD was not very large (see Fig. 2). Next, our experimental protocol included two tasks. We expected that children with ASD would be better able to produce FEs in the imitation task than in the on request task through the help of a model. However, children’s productions were better in the on request task. As for TD children alone [6], the inclusion of a group of children with ASD did not modify the model. Children (TD or ASD) tend to exactly copy the avatar, producing FEs without searching for which emotion they have to convey. Indeed, FEs are less credible than they are in the on request task. These results are in line with what was found in adults with ASD [23]. These adults were better at producing FEs when they were explicitly asked to convey an emotion than in a standard posed task. However, these results are not in agreement with the observation of Loveland et al. [20], who found no difference between an imitation task and an expression task in terms of FE production in children with ASD. Finally, we found a significant effect for centre location in favour of children from Nice when we added the children with ASD to the model, as we found when examining only the TD children [6]. These findings also concur with the literature and the well-known effect of social environment and culture on FE production [42].

In contrast to our hypothesis, we found no significant effect of age on the production of FEs in the multivariate model run on children with ASD and TD children even if the productions of older children tended to have better ratings. However, when we added IQ to the multivariate model that we applied only to children with ASD, we found no effect of age but a significant effect of the IQ on the quality of children’s FE productions. Children with ASD and a lower IQ produced FEs with less accuracy than children with ASD and a normal IQ. These observations are congruent with the data in the literature [14]. We could not explore age and IQ in the main model applied to both TD children and children with ASD because IQ was not measured in TD children. Therefore, interpretations should be proposed with caution. First, it is possible that when children with ASD and a lower IQ are included, developmental age is more important than chronological age. Second, children with ASD produce FEs in an odd way and are less able to spontaneously learn the correct way to produce FEs. Some authors have hypothesized that this difficulty comes from a lack of spontaneous imitation in children with ASD that could prevent them from naturally learning how to produce correct FEs (see [43] for a review). Finally, we found no effect of gender.

### Evaluation of FEs with vision computing

We also aimed to characterize FE production in children with ASD with an FE recognition algorithm. Based on previous works in computer vision [44], we successfully combined facial landmarks and feature extraction with a random forest classifier. The accuracy reached when testing FEs of TD children after training on TD children were very good to excellent, except for sadness, which is a FE that is still difficult to produce in children in an experimental context. When testing FEs of children with ASD, RF classifiers yielded excellent accuracy only for happiness when trained on TD children. The accuracy of all other combinations was significantly lower. These results confirmed the results of the behavioural annotations. We conducted several control calculations to assess possible biases (gender, age, city origin, size of the database for training) that confirm the robustness of our results.

More interestingly, we were able to determine which features were relevant for classifying FEs. First, the RF classifiers used both types of features to achieve the best classifications. Compared to appearance features (HOG), distance features appeared to predominantly contribute to RF classification in both groups. Leo et al. also found that HOG were important contributing features using a machine learning algorithm [31]. We chose to combine HOG and distance features as distance features were recently used to successfully explore FEs using motion capture during an imitation task in children with ASD [29]. However, no study used both features to explore relative contributions. Second, when we explored the dynamics of the classification between groups, it appears that RF classifiers needed more facial landmarks to achieve FE classification in children with ASD than in TD children. This observation was replicated with the FEs of joy, anger and neutral even when we trained the RF classifiers on a sub-dataset of TD children matched to children with ASD in age, gender and culture or when we controlled for differences in sample size. This finding supports the idea that FE productions in children with ASD are more ambiguous and, as a consequence, harder to classify [15, 16, 20]. We hypothesize that the impression of oddity described by human judges comes from this ambiguity. These results concur with the findings of Zane et al., who found worse valence predictability in children with ASD, suggesting more ambiguous expressions [29]. The difficulty in classifying FEs in children with ASD can also come from the variance in their productions that seem to be larger than that in TD children. Additionally, training RF classifiers on children with ASD does not increase the accuracy of the classification of FEs in children with ASD, supporting the fact that people with ASD do not share a specific way to produce FEs [21].

In the literature, it has been shown that children with ASD tend to focus on the mouth instead of the eyes when they explore a human face [33]. Although producing a FE with emotional valence is not the same as exploring a face, we did not observe a specific use of the mouth or neglected use of the eyes (data not shown). This observation concurs with the findings that even the abilities of children with ASD to correctly produce the upper or the lower face configuration depend on emotion subtypes, and they mostly depend on the children who produced the FEs [31], meaning that variability is higher in ASD children, as shown previously [27]. As shown in Fig. 2 and Additional file 1: Figures S1–S6, the RF classifiers use roughly the same facial landmarks in children with ASD and in TD children, but they need more facial landmarks to achieve the best classification for children with ASD. This more ambiguous way of producing FEs in children with ASD is also revealed by the way RF classifiers made mistakes. For example, we observed that when the algorithm was trained on TD children, it tended to classify anger as joy more often when it was testing children with ASD (18.62%) than TD children (5.18%). However, we believe that the proposal of Grossman and Narayanan to use measures of synchrony and complexity would be the next step for our dataset to explore their preliminary findings [27, 28].

## Limitations

Despite its novelty, our study has some limitations. First, to collect a large database of TD children to produce high-performing FE classifiers, we did not measure IQ in TD children. This prevents us from using IQ in models exploring an interaction with age. In addition, the ASD group had slightly lower IQ than the average; this might have exaggerated the differences between the two groups, as IQ has the potential to influence FE production [14]. Additionally, we found that shorter videos tended to be better rated than longer videos, but this effect was not significant. Future research should control the duration of the videos to better understand the importance of this factor. Second, compared to the large dataset of TD children, our group of children with ASD was modest in terms of sample size. However, we tried to compensate for this limitation by using several control calculations that were made possible by the high accuracy of our RF classifiers. When we lowered the sample size of the TD children, recognition accuracy for sadness decreased, indicating that the number of videos influences the accuracy of the algorithm. However, we did not find changes in the results for joy, anger and neutral, meaning that the impact of sample size was limited. Third, we adopted two experimental protocols to help children produce FEs. We do not know whether our results would have been similar using more natural scenarios [14, 15]. Fourth, annotations by human judges were made on the entire video, using audio cues when available, while the RF classifiers used only the apex of the FE to classify it, making the two annotations difficult to compare. Future research should compare audio-visual human judgement with audio and visual computing classifiers. However, despite these differences in annotation, FEs of children with ASD appear to be more difficult to classify in both situations, supporting that their productions are more ‘awkward’ than the productions of TD children [20]. Furthermore, the judges were not blinded to the diagnosis, which could have influenced their ratings. Fifth, we did not assess social skills in either group despite social skills and facial expressiveness being related [45]. Finally, we did not take into account factors that could influence the classification accuracy of RF classifiers, such as the type of tasks and the facial characteristics of children (e.g. origin and gender). Future studies should explore the impact of these factors.

## Conclusion

FE production in children with ASD has received less attention than their capacities in FE recognition. In the current study, we used two parallel approaches: human judge ratings and computer vision methods. The first approach, which took into account many confounding factors, demonstrated that children with ASD indeed have more difficulty producing recognizable and credible FEs. The second approach yielded that the production of FEs in children with ASD carries more ambiguity, as shown by the fact that RF classifiers needed more facial landmarks to classify FE production in children with ASD or tended to classify anger as joy more often in children with ASD. More research is needed to better characterize deficits in FE production in children with ASD. We believe that the combination of more ecological frameworks for FE production and computer vision would be a possible next step to better understand FE production in children with ASD.

## Supplementary information

**Additional file 1.** **Table S1.** Matching characteristics of the TD subgroups for machine learning. **Table S2.** Emotion production in children with ASD as a function of age, gender, group, order, modality, elicitation task, emotion and sites: results from the GLMM model. **Table S3.** Durations of videos during emotion production in children with ASD and TD children. **Table S4.** Radom forest classifier accuracy recognition of the FE. **Table S5.** Radom forest classifier accuracy recognition of FE when learning on TD group 1 or 2. **Table S6.** Confusion matrix when learning and testing on TD. **Table S7.** Confusion matrix when learning on TSA and testing on TSA. **Table S8.** Confusion matrix when learning on TD and testing on TSA. **Table S9.** Confusion matrix when learning on TD1 and testing on TD1. **Table S10.** Confusion matrix when learning on TD2 and testing on TD2. **Figure S1.** Histogram of the videos’ duration. **Figure S2** and **S3.** Facial landmarks contributing to classification of anger using random forest (training and testing) in children with typical development (left) and children with autism spectrum disorder (S2: Distance; S3: HOG). **Figure S4** and **S5.** Facial landmarks contributing to classification of neutral facial expression using random forest (training and testing) in children with typical development (left) and children with autism spectrum disorder (S4: Distance; S5: HOG). **Figure S6** and **S7.** Facial landmarks contributing to classification of sadness using random forest (training and testing) in children with typical development (left) and children with autism spectrum disorder (S6: Distance; S7: HOG).

## Data Availability

The datasets used and/or analysed during the current study are available from the corresponding author on reasonable request.

## Abbreviations

ADI-R: Autism Diagnosis Interview-Revised
ADOS: Autism Diagnosis Observation Schedule
ASD: Autism spectrum disorders
FE: Facial expression
GLMM: Generalized linear mixed models
HOG: Histogram of oriented gradients
IOD: Inter-ocular distance
IQ: Intellectual quotient
RF: Random forest
TD: Typical development
