# Supplementary material for: Children with autism spectrum disorder produce more ambiguous and less socially meaningful facial expressions: an experimental study using random forest classifiers
Source: Mol Autism. 2020 Jan 13;11:5. doi: 10.1186/s13229-020-0312-2 (PMC6958757; doi:10.1186/s13229-020-0312-2)

**Additional file**

**Grossard et al.**

| **Table S1. Matching characteristics of the TD subgroups for machine learning** | | | | |
| --- | --- | --- | --- | --- |
|  |  | **ASD** | **TD group 1** | **TD group 2** |
| Neutral | N _videos_ | 125 | 138 | 150 |
|  | % | 37.65 | 36.03 | 34.56 |
| Joy | N _videos_ | 101 | 109 | 118 |
|  | % | 30.42 | 28.45 | 27.18 |
| Anger | N _videos_ | 58 | 84 | 103 |
|  | % | 17.46 | 21.93 | 23.73 |
| Sadness | N _videos_ | 45 | 52 | 63 |
|  | % | 13.55 | 13.57 | 14.51 |
| City | Paris | 15 | 15 | 15 |
|  | Nice | 19 | 19 | 19 |
| School | Preschooler | 5 | 5 | 5 |
|  | Grade 1 | 3 | 3 | 3 |
|  | Grade 2 | 7 | 7 | 7 |
|  | Grade 3 | 8 | 9 | 8 |
|  | Grade 4 | 9 | 10 | 11 |
|  | Grade 5 | 1 | 0 | 0 |
|  | Grade 6 | 1 | 0 | 0 |
| Gender | Male | 26 | 26 | 26 |
|  | Female | 8 | 8 | 8 |
| Total | N _children_ | 34 | 34 | 34 |
|  | N _videos_ | 332 | 383 | 434 |

| **Table S2. Emotion production in children with ASD as a function of age, gender, group, order, modality, elicitation task, emotion and sites: results from the GLMM model** | | | |
| --- | --- | --- | --- |
| Variable | ß estimate | Standard error | p |
| Age | - 0.055 | 0.107 | 0.61 |
| Gender (boys vs. girls) | -0.381 | 0.367 | 0.299 |
| Order 2 vs. order 1 | 0.569 | 0.424 | 0.18 |
| Order 3 vs. order 1 | -0.614 | 0.481 | 0.201 |
| Order 4 vs. order 1 | -0.027 | 0.422 | 0.949 |
| Modality (visual vs. audiovisual) | 0.146 | 0.162 | 0.37 |
| Elicitation task (on request vs. imitation) | 0.574 | 0.176 | 0.001 |
| Emotion (happiness vs. sadness) | 1.609 | 0.236 | <0.001 |
| Emotion (neutral vs. sadness) | 1.481 | 0.233 | <0.001 |
| Emotion (anger vs. sadness) | 0.678 | 0.216 | 0.001 |
| Site (Nice vs. Paris) | - 0.603 | 0.323 | 0.062 |
| IQ | 0.031 | 0.01 | 0.002 |

The GLMM formula was the following: score ~ age + gender + order + task + modality + emotion subtype + center + IQ + (1|child number).

| **Table S3. Durations of videos during emotion production in children with ASD and TD children** | | | | |
| --- | --- | --- | --- | --- |
|  | Estimate | CI95_low | CI95_up | p value |
| (Intercept) | 76.06 | 71.18 | 80.95 | < 0.001 |
| Modality (visual vs. audiovisual) | 8.21 | 5.69 | 10.66 | < 0.001 |
| Emotion (anger vs. sadness) | -21.59 | -25.07 | -18.13 | < 0.001 |
| Emotion (happiness vs. sadness) | -16.22 | -19.71 | -12.76 | < 0.001 |
| Emotion (neutral vs. sadness) | -2.02 | -5.48 | 1.52 | 0.26 |
| Elicitation task (on request vs. imitation) | 8.32 | 5.64 | 10.88 | < 0.001 |
| Group (TD vs. ASD) | 30.76 | 25.69 | 35.93 | < 0.001 |

The GLMM formula was the following: video duration ~ task + modality + emotion subtype + group + (1|child number).

In average, facial expressions had:

- A longer duration for the unimodal condition (8 ms more compared to the multimodal condition)
- A shorter duration for anger than sadness (Δ=-22 ms)
- A shorter duration for joy than sadness (Δ=-16 ms)
- A longer duration for the ‘on request task’ than the ‘imitation task’ (Δ=+8 ms)
- A longer duration for TD children than ASD (Δ=+31 ms)

**Figure S1. Histogram of the videos’ duration**


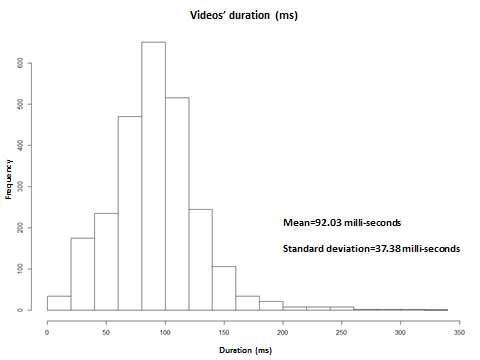


| **Table S4. Radom forest classifier accuracy recognition of the FE** | | | | |
| --- | --- | --- | --- | --- |
| Learning on | TDall-(TD group 1)  (N=126) | TDall-(TD group 2)  (N=126) | TDall-(TD group 1)  (N=126) | TDall-(TD group 2)  (N=126) |
| Testing | TD group 1  (N=34) | TD group 2  (N=34) | ASD  (N=34) | ASD  (N=34) |
| Neutral | 82.61 | 82.0 | 67.2 | 67.2 |
| Happiness | 88.99 | 90.52 | 88.12 | 87.13 |
| Anger | 84.52 | 80.81 | 58.62 | 63.79 |
| Sadness | 61.54 | 62.3 | 48.89 | 40.0 |
| Global accuracy | 81.98 | 81.22 | 69.6 | 69.0 |

| **Table S5. Radom forest classifier accuracy recognition of FE when learning on TD group 1 or 2** | | |
| --- | --- | --- |
| Learning on | TD group 1  (N=34) | TD group 2  (N=34) |
| Testing | TD group 1  (N=34) | TD group 2  (N=34) |
| Neutral | 82.30 | 82.87 |
| Happiness | 87.76 | 87.76 |
| Anger | 76.91 | 76.91 |
| Sadness | 42.78 | 29.01 |

| **Table S6. Confusion matrix when learning and testing on TD** | | | | |
| --- | --- | --- | --- | --- |
|  | Neutral | Happiness | Anger | Sadness |
| Neutral | 86.64 | 3.43 | 6.09 | 3.84 |
| Happiness | 5.06 | 90.47 | 1.64 | 2.83 |
| Anger | 10.39 | 5.18 | 79.76 | 4.67 |
| Sadness | 23.24 | 9.01 | 11.5 | 56.15 |

| **Table S7. Confusion matrix when learning on TSA and testing on TSA** | | | | |
| --- | --- | --- | --- | --- |
|  | Neutral | Happiness | Anger | Sadness |
| Neutral | 72.55 | 9.11 | 13.27 | 5.07 |
| Happiness | 12.26 | 70.38 | 14.02 | 6.14 |
| Anger | 21.25 | 17.25 | 58.17 | 10.52 |
| Sadness | 15.83 | 9.76 | 32.62 | 44.44 |

| **Table S8. Confusion matrix when learning on TD and testing on TSA** | | | | |
| --- | --- | --- | --- | --- |
|  | Neutral | Happiness | Anger | Sadness |
| Neutral | 68.08 | 10.32 | 16.88 | 4.72 |
| Happiness | 6.93 | 85.05 | 1.88 | 6.14 |
| Anger | 12.24 | 18.62 | 58.62 | 10.52 |
| Sadness | 22.67 | 9.78 | 23.11 | 44.44 |

| **Table S9. Confusion matrix when learning on TD1 and testing on TD1** | | | | |
| --- | --- | --- | --- | --- |
|  | Neutral | Happiness | Anger | Sadness |
| Neutral | 82.30 | 2.53 | 9.16 | 6.01 |
| Happiness | 3.54 | 87.76 | 1.00 | 7.70 |
| Anger | 13.58 | 7.43 | 76.91 | 2.08 |
| Sadness | 30.39 | 13.17 | 13.67 | 42.78 |

| **Table S10. Confusion matrix when learning on TD2 and testing on TD2** | | | | |
| --- | --- | --- | --- | --- |
|  | Neutral | Happiness | Anger | Sadness |
| Neutral | 82.87 | 3.58 | 9.55 | 4.00 |
| Happiness | 5.49 | 87.87 | 0.00 | 6.64 |
| Anger | 13.63 | 7.43 | 75.09 | 3.84 |
| Sadness | 27.78 | 11.97 | 31.25 | 29.01 |

**Figure S2 and S3. Facial landmarks contributing to classification of anger using random forest (training and testing) in children with typical development (left) and children with autism spectrum disorder (S2: Distance; S3: HOG)**


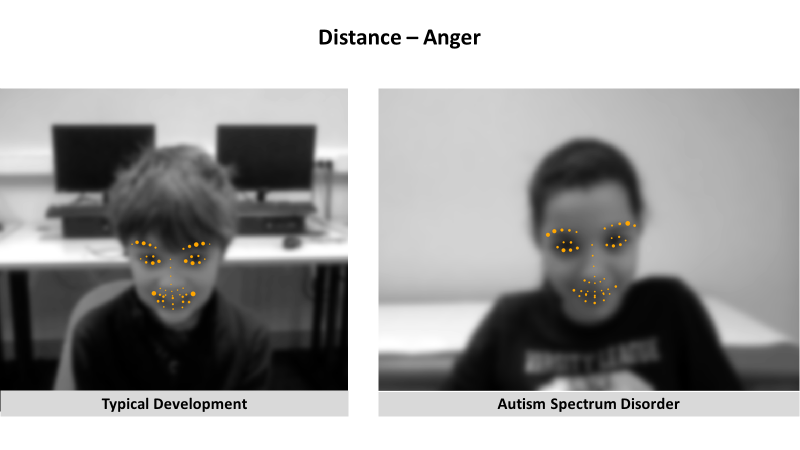


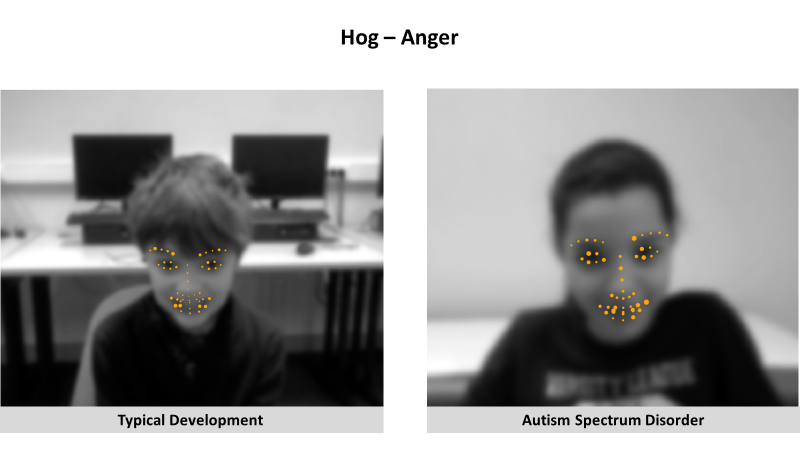


**Figure S4 and S5. Facial landmarks contributing to classification of neutral facial expression using random forest (training and testing) in children with typical development (left) and children with autism spectrum disorder (S4: Distance; S5: HOG)**


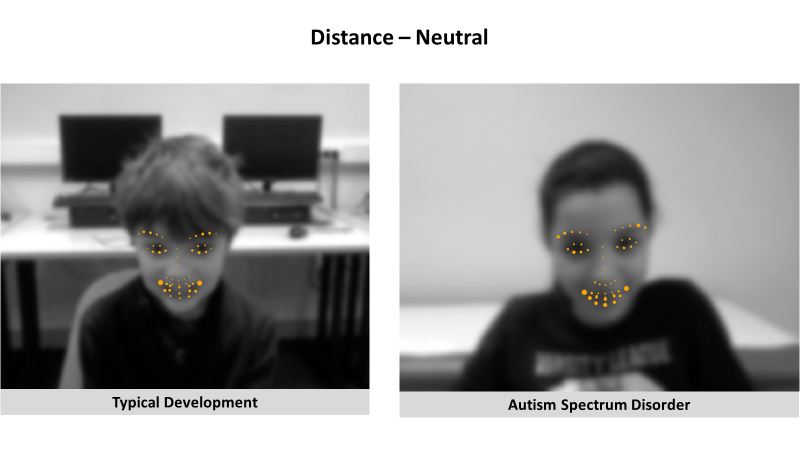


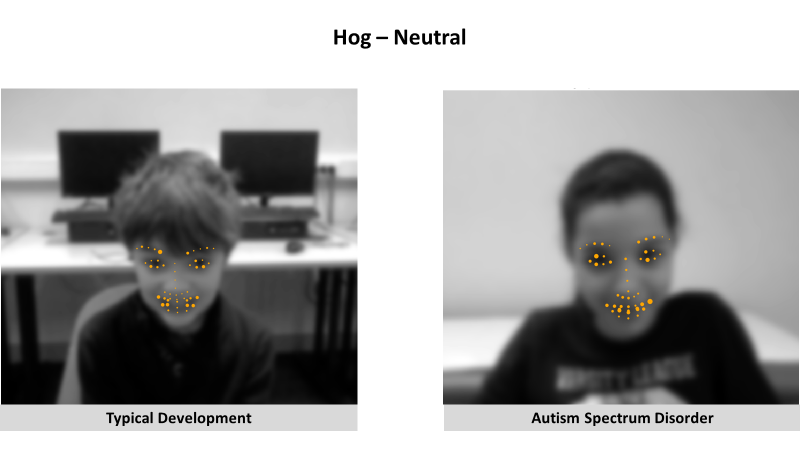


**Figure S6 and S7. Facial landmarks contributing to classification of sadness using random forest (training and testing) in children with typical development (left) and children with autism spectrum disorder (S6: Distance; S7: HOG)**


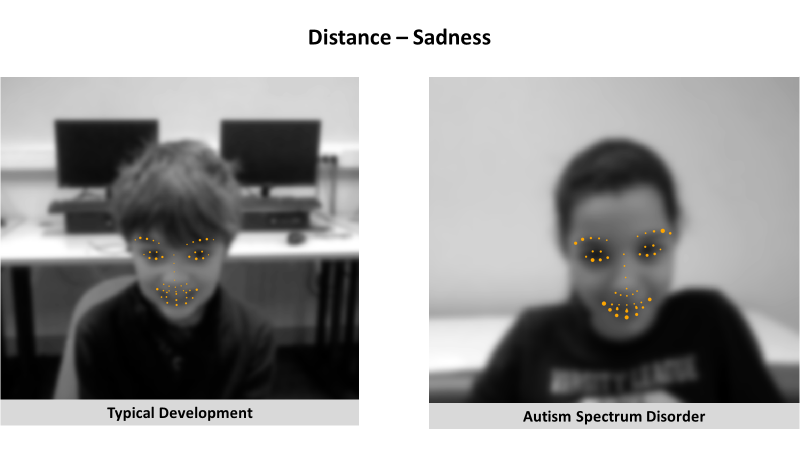


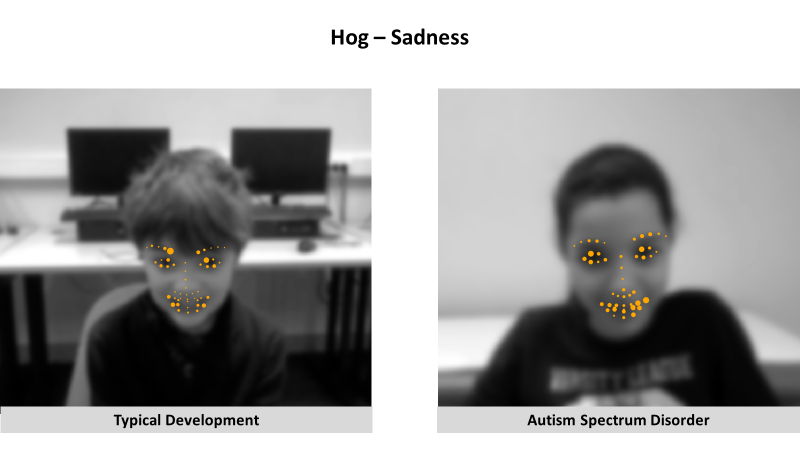

Supplement: Supplementary file 1 — Additional file 1. Table S1. Matching characteristics of the TD subgroups for machine learning. Table S2. Emotion production in children with ASD as a function of age, gender, group, order, modality, elicitation task, emotion and sites: results from the GLMM model. Table S3. Durations of videos during emotion production in children with ASD and TD children. Table S4. Radom forest classifier accuracy recognition of the FE. Table S5. Radom forest classifier accuracy recognition of FE when learning on TD group 1 or 2. Table S6. Confusion matrix when learning and testing on TD. Table S7. Confusion matrix when learning on TSA and testing on TSA. Table S8. Confusion matrix when learning on TD and testing on TSA. Table S9. Confusion matrix when learning on TD1 and testing on TD1. Table S10. Confusion matrix when learning on TD2 and testing on TD2. Figure S1. Histogram of the videos’ duration. Figure S2 and S3. Facial landmarks contributing to classification of anger using random forest (training and testing) in children with typical development (left) and children with autism spectrum disorder (S2: Distance; S3: HOG). Figure S4 and S5. Facial landmarks contributing to classification of neutral facial expression using random forest (training and testing) in children with typical development (left) and children with autism spectrum disorder (S4: Distance; S5: HOG). Figure S6 and S7. Facial landmarks contributing to classification of sadness using random forest (training and testing) in children with typical development (left) and children with autism spectrum disorder (S6: Distance; S7: HOG). [file 13229_2020_312_MOESM1_ESM.docx]
